# Correction of metabolic acidosis improves insulin resistance in chronic kidney disease

**DOI:** 10.1186/s12882-016-0372-x

**Published:** 2016-10-22

**Authors:** Antonio Bellasi, Lucia Di Micco, Domenico Santoro, Stefania Marzocco, Emanuele De Simone, Mario Cozzolino, Luca Di Lullo, Pasquale Guastaferro, Biagio Di Iorio, on behalf of UBI study investigators, Simona Adesso, Annamaria Bruzzese, Giuseppe Conte, Adamasco Cupisti, Antonella De Blasio, Alfonso Frallicciardi, Rachele Grifa, Rosa Martino, Matteo Piemontese, Maria Luisa Sirico, Giuseppe Struzziero, Raffaele Tortoriello, Fabio Vitale

**Affiliations:** 1 Department of Nephrology and Dialysis, ASST-Lariana, Ospedale Sant’ Anna, Como, (CO) Italy; 2 Department of Nephrology and Dialysis, UOC Nefrologia, PO “A Landolfi”, Via Melito, snc, I-83029, Solofra, (AV) Italy; 3 Department of Clinical and Experimental Medicine, Unit of Nephrology, University of Messina, Messina, Italy; 4 Department of Pharmacy, School of Pharmacy, University of Salerno, Fisciano, (SA) Italy; 5 Department of Health Sciences, University of Milan, Milan, Italy; 6 Department of Nephrology and Dialysis, Ospedale Parodi, Delfino, Colleferro, (Rome) Italy; 7 Dialysis, Sant’Angelo dei Lombardi, Avellino Italy

**Keywords:** CKD, Diabetes, Metabolic acidosis, Homa-test, Sodium bicarbonate

## Abstract

**Background:**

Correction of metabolic acidosis (MA) with nutritional therapy or bicarbonate administration is widely used in chronic kidney disease (CKD) patients. However, it is unknown whether these interventions reduce insulin resistance (IR) in diabetic patients with CKD. We sought to evaluate the effect of MA correction on endogenous insulin action in diabetic type 2 (DM2) CKD patients.

**Methods:**

A total of 145 CKD subjects (83 men e 62 women) with DM2 treated with oral antidiabetic drugs were included in the study and followed up to 1 year. All patients were randomly assigned 1:1 to either open-label (A) oral bicarbonate to achieve serum bicarbonate levels of 24–28 mmol/L (treatment group) or (B) no treatment (control group). The Homeostatic model assessment (HOMA) index was used to evaluate IR at study inception and conclusion. Parametric and non-parametric tests as well as linear regression were used.

**Results:**

At baseline no differences in demographic and clinical characteristics between the two groups was observed. Average dose of bicarbonate in the treatment group was 0.7 ± 0.2 mmol/kg. Treated patients showed a better metabolic control as confirmed by lower insulin levels (13.4 ± 5.2 vs 19.9 ± 6.3; for treated and control subjects respectively; *p* < 0.001), Homa-IR (5.9[5.0-7.0] vs 6.3[5.3–8.2]; *p* = 0.01) and need for oral antidiabetic drugs. The serum bicarbonate and HOMA-IR relationship was non-linear and the largest HOMA-IR reduction was noted for serum bicarbonate levels between 24 and 28 mmol/l. Adjustment for confounders, suggests that serum bicarbonate rather than treatment drives the effect on HOMA-IR.

**Conclusions:**

Serum bicarbonate is related to IR and the largest HOMA-IR reduction is noted for serum bicarbonate between 24 and 28 mmol/l. Treatment with bicarbonate influences IR. However, changes in serum bicarbonate explains the effect of treatment on HOMA index. Future efforts are required to validate these results in diabetic and non-diabetic CKD patients.

**Trial registration:**

The trial was registered at www.clinicaltrial.gov (Use of Bicarbonate in Chronic Renal Insufficiency (UBI) study - NCT01640119)

## Background

Incidence of chronic kidney disease (CKD) as well as the prevalence of diabetic subjects among CKD patients are steadily increasing [1, 2]. As renal function declines, metabolic acidosis and insulin resistance (IR) commonly arise. Among others, these metabolic complications are associated with serious consequences on bones and nutritional status [3, 4] and likely contribute to some of the abysmal risk of death associated with CKD.

Insulin resistance (IR) is characterized by suboptimal biological responses of the liver, skeletal muscle and adipose tissue to normal amounts of insulin secreted [4]. Conditions such as metabolic acidosis, anemia, inflammation, hyperactivity of the Renin-Angiotensin-Aldosterone System (RAAS), vitamin D deficiency, physical inactivity, excess of fat mass as well as nitrogen catabolites accumulation have all been implicated in IR in CKD subjects [5]. Notably, several clinical consequences have been linked to IR. Indeed, IR may promote endothelial dysfunction and portends increased cardiovascular mortality. Although evidence is not conclusive, some data also suggest that IR is a harbinger of CKD incidence and progression. Based on these lines of evidence, it is conceivable that IR represents a modifiable risk factor and a potential therapeutic target to improve CKD outcome [4–6].

The association between metabolic acidosis, IR and the cardiovascular risk has been documented in the scientific literature since 1924 [7]. However, in spite of the fact that correction of metabolic acidosis with nutritional therapy and/or oral administration of sodium bicarbonate in CKD is widely used [8–10], it is unknown whether correction of metabolic acidosis reduces IR and/or improves insulin effects on target cells in diabetic subjects.

We aim to evaluate whether metabolic acidosis correction by sodium bicarbonate administration may improve peripheral endogen insulin utilization by target organs in diabetic subjects with CKD treated with oral antidiabetic drugs.

## Methods

For current analyses, we analyzed the first 145 subjects (83 men and 62 women) with Diabetes Mellitus type 2 not treated with insulin participating in the Use of Bicarbonate in Chronic Renal Insufficiency (UBI) study (NCT NCT01640119) with at least 1 year of follow-up. The UBI study protocol has been published previously [11]. Briefly, the UBI study is an on-going multi-center, open-label, randomized controlled study designed to test the impact of metabolic acidosis correction on CKD progression to End Stage Renal Disease (ESRD). CKD-3b-4 patients of 18 to 80 year of age, able to provide written informed consent and serum bicarbonate levels below 24 mEql/l are randomized (allocation ratio 1:1) to either oral sodium bicarbonate (treatment group) or conventional therapy for CKD (control group). Study investigators are free to adjust medications to achieve the targets for glycated hemoglobin, bone mineral metabolism, blood pressure, anemia, iron status, dyslipidemia as suggested by guidelines on CKD patients’ management available at the time of the study design [11]. The randomization process is centralized to ensure allocation concealment. Patients with evidence of neoplastic diseases, autoimmune diseases, chronic heart failure NYHA class III-IV, uncontrolled arterial hypertension, severe peripheral arterial disease (defined as limb amputation), cerebrovascular disease, neobladder or ureterosigmoidostomy, severe metabolic acidosis (defined as serum bicarbonate <18 mEq/l) or use of calcium carbonate in the 3-month prior to study inclusion are excluded from the trial. Oral sodium bicarbonate is administered at the dose of 0.5 mmol/kg of body weight (1 g of sodium bicarbonate contains 11.9 mmol – initial dose about 3–4 g) two times a day until the achievement of the desired serum bicarbonate target of 24–28 mmol/l. If a serum value of 28 mmol/l is exceeded, the administration of bicarbonate is tapered each 3 days until the desired serum target level is achieved [11].

### Demographic, clinical and laboratory characteristics

Demographic and clinical characteristics were assessed as study inception. Self-reported variables included age, sex. Medical chart reviews were conducted to determine the presence of diabetes mellitus status or the use of oral antidiabetic medications, history of atherosclerotic cardiovascular disease (ASCVD) and the use of different medications. History of ASCVD was a composite measure that included myocardial infarction, angina, and peripheral and cerebrovascular disease. Blood pressure was measured after a 15 to 20 min rest, using a manual aneroid sphygmomanometer.

Routine biochemical laboratory measurements were obtained at baseline and completion 12 months of follow-up and analyzed at the facilities usual laboratories as part of the standard patients care. All blood samples were in a fasting condition. Insulin resistance was evaluated via the Homeostatic Model Assessment (HOMA) test at baseline and at completion of 12 months of follow-up.

Finally, 25-OH vitamin D was measured every 3 months; the correction of low levels was started at values lower than 20 ng/ml and stopped at values higher than 50 ng/ml.

Patients using steroids and other drugs interfering directly with glucose levels were excluded from the study.

### Insulin resistance measurement and HOMA test

Insulin resistance was assessed indirectly by the Homeostatic model assessment (HOMA) index as suggested by Wallace and coworkers [12]. Briefly, the HOMA index is a mathematical model that allows to calculate insulin sensitivity (HOMA-IR) and evaluate ß pancreatic cell function (HOMA-%B) from fasting plasma glucose and insulin levels [12]. It is a simple test, appropriate to perform in large epidemiological studies that nicely correlates with experimental data obtained with direct measurement techniques such as the euglycemic clamp [13–16].

To perform the HOMA test, blood samples are drawn twice (30 min apart) in 3 consecutive days. Patients are kept at rest, in a fasting status for at least 8 h before the blood sampling. Tobacco use is forbidden for the 12 h before blood tests. The presented values for HOMA test at baseline and study completion are the mean values of the three consecutive blood samples. For HOMA-IR and HOMA-%B calculation, the following formulas are used [12]:

- HOMA-IR = (FPI * FPG)/22.5;
- HOMA-%B = (20 * FPI)/(FPG - 3.5)

where FPI stands for fasting plasma insulin concentration (mU/l) and FPG stands for fasting plasma glucose (mmol/l) (FPG conversion factor from mg/dl to mmol/l: 10.018).

HOMA-IR estimates of insulin resistance. Normal values are <0.25. Values greater or equal than 5.5 indicate insulin resistance typical of early stages of Diabetes Mellitus. HOMA-B% estimates ß pancreatic cells function. It’s value ranges from 0 % (no pancreatic cell function) to 100 % (all pancreatic cell functioning). FPI and FPG measurements were performed centrally at P.O. “A Landolfi” – Solofra (AV), Italy, via COBAS 6000 or COBAS C 501 (Roche Diagnostics) and IMMULITE 2000 (Siemens Healthcare Global), respectively.

### Study objective and endpoint

Current analyses aim at testing the impact of metabolic acidosis correction in CKD 3b-4 diabetic patients with serum bicarbonate <24 mEq/l on insulin resistance evaluated via the Homeostatic Model Assessment (HOMA) test. The HOMA was performed at study inception and after 12 months of treatment with either oral sodium bicarbonate (treatment group) or conventional therapy for CKD (control group).

### Statistical analysis

Data are reported as mean ± SD or counts (percentage) when appropriate. Un-paired *T*-test and Chi-square test were used to assess difference between study groups at baseline and study completion (Tables 1 and 2). The bagplot (Fig. 1) was used to describe the bivariate association of serum bicarbonate and HOMA test in subjects randomized to oral sodium bicarbonate (treated) or conventional therapy (controls) at study inception and completion. Because of the random allocation to treatment groups, the selection criterion was independent of study investigators’ beliefs (i.e., we analyzed data of the first 145 diabetic type 2 patients randomized in the UBI study who completed 1 year of follow-up) and the the optimal balance between groups at study inception, the Wilcoxon rank sum test was used to assess between- and within-group (treated vs control subjects) differences in HOMA-IR and HOMA-%B at study inception as well as completion of 12 months of follow-up (Table 3). Linear regression was used to assess the independent association of treatment and/or metabolic acidosis correction and HOMA test at study completion. First, we tested for the unadjusted association of (i) treatment allocation, (ii) serum bicarbonate values at follow-up and (iii) changes of serum bicarbonate (serum bicarbonate at follow-up – serum bicarbonate at study inception) with HOMA-IR (Table 4). Subsequently, we tested the independent contribution of metabolic acidosis correction (i.e., serum bicarbonate at study completion or changes in serum bicarbonate) vs oral bicarbonate supplementation, forcing both variables in the same regression model (Table 4). However, due to the non-linear relationship between serum bicarbonate (Fig. 2a) or changes in serum bicarbonate (Fig. 2b) and HOMA index at study completion, we tested for an interaction effect of treatment and values of serum bicarbonate at study completion or changes of serum bicarbonates (Table 4). Because of the significant effect modification of serum serum bicarbonate levels on treatment effect on HOMA test and because at visual inspection (Fig. 2a) the association between serum bicarbonate and HOMA test was different for values greater than 28 mmol/l, we performed some additional analyses by applying regression splines with a knot set at serum bicarbonate level of 28 mEq/l and tested for the independent association between serum bicarbonate, treatment and HOMA test at study completion (Table 5). All analyses were conducted as *intention-to-treat*. Two-tailed probability values ≤ 0.05 were considered statistically significant. Analyses were completed using R version 3.1.3 (2015-03-09) (The R Foundation for Statistical Computing).

**Table 1:** Demographic, clinical, laboratory characteristics and use of oral anti-diabetic medications of patients randomized to oral sodium bicarbonate (Treated) or conventional therapy (controls) at study inception

	Overall	Treated	Control	*p*-value
	(*N* = 145)	(*N* = 71)	(*N* = 74)	
Males, N (%)	83 (57 %)	47 (66 %)	36 (48 %)	NS
Age, years	65.5 ± 11.4	64.9 ± 11.8	66.0 ± 12.9	NS
Body Weight, kg	75.5 ± 14.1	76.5 ± 14.6	73.4 ± 11.2	NS
Cardiovascular disease, N(%)	36 (25)	17 (24)	19 (26)	NS
Systolic blood pressure, mmHg	122 ± 20	124 ± 19	120 ± 22	NS
Disatolic blood pressure, mmHg	73 ± 9	73 ± 8	73 ± 10	NS
Serum Bicarbonate, mEql/l	21.4 ± 1.9	21.2 ± 1.9	21.6 ± 2.0	NS
Serum Gucose, mg/dl	150 ± 44	149 ± 41	151 ± 47	NS
HbA1C %	6.76 ± 1.2	6.74 ± 1.0	6.8 ± 1.4	NS
Serum creatinine,mg/dl	2.1 ± 0.8	2.3 ± 0.8	2.0 ± 0.7	NS
BUN, mg/dl	87 ± 32	93 ± 35	81 ± 28	NS
Creatinine clearance, ml/min	33 ± 14	32 ± 14	35 ± 15	NS
Uric Acid, mg/dl	5.4 ± 1.8	5.6 ± 1.9	5.1 ± 1.8	NS
Serum sodium, mEql/l	139 ± 3	139 ± 3	139 ± 2	NS
Serum potassium, mEq/l	4.82 ± 0.7	4.85 ± 0.6	4.79 ± 0.7	NS
Total serum calcium, mg/dl	9.13 ± 0.6	9.14 ± 0.62	9.12 ± 0.58	NS
Serum phosphate, mg/dl	3.7 ± 0.7	3.8 ± 0.7	3.7 ± 0.7	NS
Serum albumin, g/dl	3.86 ± 0.42	3.85 ± 0.39	3.89 ± 0.46	NS
Hemoglobin, g/dl	12.3 ± 1.7	12.26 ± 1.82	12.39 ± 1.68	NS
C-Reactive Protein, mg/l	11.20 ± 28.1	11.08 ± 34.37	11.34 ± 18.53	NS
Serum PTH, pg/ml	122 ± 83	119 ± 34	124 ± 88	NS
Serum total cholesterol, mg/dl	154 ± 34	158 ± 34	151 ± 33	NS
Serum LDL cholesterol, mg/dl	91 ± 32	93 ± 31	87 ± 32	NS
Serum HDL cholesterol, mg/dl	45 ± 14	45 ± 12	45 ± 16	NS
Serum triglicerides, mg/dl	134 ± 58	130 ± 56	138 ± 60	NS
vitamin D (25-OH.D), ng/ml	39 ± 11	39 ± 10	38 ± 10	NS
Homa-IR	7.17 ± 2.4	7.13 ± 2.5	7.20 ± 2.36	NS
HOMA % B	49 ± 21	50 ± 22	48 ± 21	NS
Serum insulin, mcIU	18.3 ± 6.6	17.6 ± 6.1	19.0 ± 7.0	NS
Antidiabetic medications				
Biguanides, number (%)	98 (67.5)	52 (73.2)	46 (62.2)	NS
dose, mg/day	1740 ± 417	1760 ± 611	1725 ± 670	NS
Solfonylureas, number (%)	46 (31.7)	17 (23.9)	29 (39.2)	NS
dose, mg/day	5.25 ± 1.19	5.29 ± 1.38	5.23 ± 1.14	NS
Meglitinides, number (%)	41 (28.3)	21 (29.6)	20 (27)	NS
dose, mg/day	3.13 ± 1.35	3.52 ± 0.91	2.76 ± 1.59	NS
Use of > 1 medication, number (%)	37 (25.5)	20 (28.1)	17 (23)	NS
Antihypertensive DRUGS				
Furosemide, number (%)	131 (90.3)	62 (87.3)	69 (93.3)	NS
dose, mg/day	55 ± 19	55 ± 21	55 ± 17	NS
ARB inhibitors, number (%)	75 (51.7)	37 (23.9)	38 (39.2)	NS
ACE-Inhibitors, number (%)	74 (51)	38 (52.1)	36 (48.6)	NS
Beta-blocker (%)	24 (16.5)	14 (19.7)	10 (13.5)	NS
Other antihypertensive drugs number (%)	42 (28.9)	20 (28.2)	22 (29.7)	NS
Use of > 1 medication, number (%)	70 (48.3)	38 (53.5)	32 (43.2)	NS

Continuous and dichotomous variables are expressed as mean ± standard deviation or count (%), respectively

**Table 2:** Clinical, laboratory characteristics and use of anti-diabetic medications of patients randomized to oral sodium bicarbonate (Treated) or conventional therapy (controls) at study completion

	Overall	Treated	Control	*p*-value
	145	71	74	
Body Weight, kg	76.1 ± 12.8	76.3 ± 12.8	73.4 ± 15.0	NS
Systolic blood pressure, mmHg	123 ± 17	125 ± 17	121 ± 16	NS
Disatolic blood pressure, mmHg	74 ± 8	76 ± 8	72 ± 10	NS
Serum Bicarbonate, mEql/l	24.2 ± 2.7	26.0 ± 2.0	22.3 ± 1.9	0.0001
Serum Gucose, mg/dl	118 ± 29	110 ± 32	127 ± 24	0.0001
HbA1C %	7.2 ± 2.9	6.7 ± 0.9	7.7 ± 3.7	0.028
Creatinine Clearance, ml/min	30 ± 16	32 ± 15	31 ± 16	NS
Homa-IR	6.52 ± 1.8	6.1 ± 1.5	7.0 ± 2.0	0.003
HOMA % B	52 ± 20	55 ± 18	49 ± 21	0.015
Serum insulin, mcIU	16.4 ± 6.6	13.4 ± 5.2	19.9 ± 6.3	0.0001
Antidiabetic medications				
Biguanides, number (%)	89 (61.4)	45 (63.3)	44 (59.4)	NS
dose, mg/day	1570 ± 517	1377 ± 457	1615 ± 550	0.005
Solfonylureas, number (%)	40 (27.6)	12 (16.9)	28 (37.8)	0.009
dose, mg/day	5.05 ± 1.29	4.89 ± 1.7	5.20 ± 1.07	0.033
Meglitinides, number (%)	36 (24.8)	16 (22.5)	20 (27)	NS
dose, mg/day	3.13 ± 1.35	3.52 ± 0.91	2.76 ± 1.59	0.0001
Use of > 1 medication, number (%)	28 (19.3)	12 (16.9)	16 (21.6)	NS

Continuous and dichotomous variables are expressed as mean ± standard deviation or count (%), respectively

**Fig. 1:**
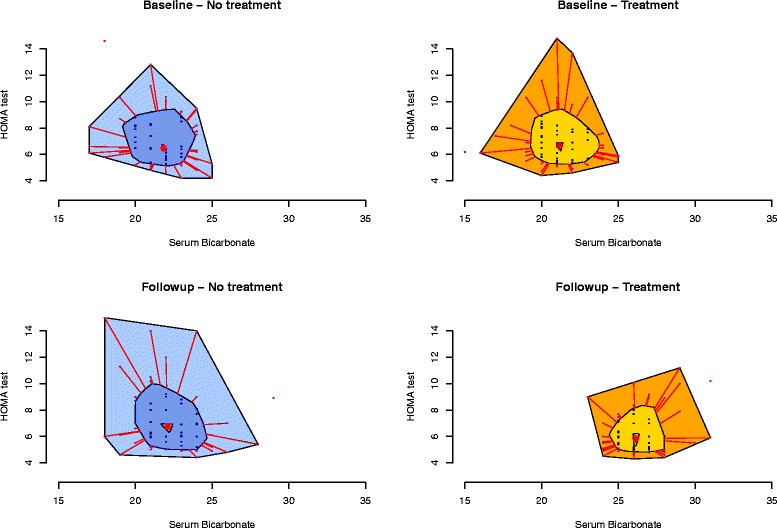
The bagplots describe the association between serum bicarbonate and HOMA test in subjects randomized to oral sodium bicarbonate (Treated) or conventional therapy (controls) at study inception and conclusion. Legend: The inner polygon (called bag) contains 50 % of all points. Observations outside the outermost polygon (called fence) are outliers. The observations between the bag and fence are marked by line segments. The asterisk symbol (*) near the center of the graph represents the bivariate median

**Table 3:** HOMA-IR and HOMA-%B at study inception and conclusion in treated and control subjects

	Treated	Control	*P* value (between group)
HOMA-IR			
Baseline	6.4 [5.5–7.9]	6.4 [5.5–8.2]	0.915
Study Completion	5.9 [5.0–7.0]	6.3 [5.3–8.2]	0.010
*P*-value (within group)	0.004	0.572	
HOMA-%B			
Baseline	50.5 [32.0–67.2]	43.0 [32.7–62.2]	0.543
Study Completion	60.5 [43.5–70.2]	45.0 [32.7–64.5]	0.023
*P*-value (within group)	0.036	0.754	

Data are expressed as median [Interquartile range]. Wilcoxon rank sum test is used for between- and within-group comparisons

**Table 4:** Predictor of HOMA index at study completion by unadjusted and multivariable adjusted linear regression analyses

Predictor of HOMA index at study completion			
Variable	B-coef	Standard Error	P value
Unadjusted			
- Treatment (yes vs no)	−0.8740	0.3285	0.0087
Unadjusted			
- Change in serum bicarbonate (%)	−1.5833	0.9462	0.0964
Unadjusted			
- Serum bicarbonate at study completion (mmol/l)	−0.14511	0.06026	0.0173
Adjusted for treatment, change in serum bicarbonate and interaction of change in serum bicarbonate*treatment			
- Treatment (yes vs no)	−1.4604	0.5015	0.00418
- Change in serum bicarbonate (%)	−3.0382	1.8007	0.09378
- Interaction test (change in serum bicarbonate*treatment)	4.9948	2.3578	0.03591
Adjusted for treatment, serum bicarbonate at follow-up and interaction of serum bicarbonate at followup*treatment			
- Treatment (yes vs no)	−11.6700	4.4255	0.00931
- Serum bicarbonate at follow-up (mmol/l)	−0.2328	0.1106	0.03713
- Interaction test (serum bicarbonate at follow-up*treatment)	0.4476	0.1784	0.01325

*interaction between factors

**Fig. 2:**
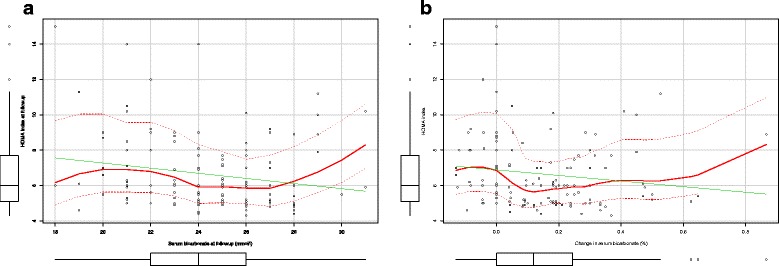
The scatterplots represent the relationship between serum bicarbonate (**a**) and changes in serum bicarbonate (**b**) and HOMA index at study completion. Legend: solid green line represents the linear regression line; solid red line represents the smoothed linear regression line

**Table 5:** Predictor of HOMA index at study completion by unadjusted and multivariable adjusted spline regression analyses

Predictor of HOMA index at study completion (further elaborations)			
Variable	B-coef	Standard Error	*P* value
Unadjusted			
- Serum bicarbonate <28 mmol/l at follow-up	−4.6008	1.1804	0.00015
- Serum bicarbonate ≥28 mmol/l at follow-up	1.9360	1.0270	0.06146
Adjusted for treatment, serum bicarbonate greater/equal or lower than 28 mmol/l			
- Treatment (yes vs no)	−0.3482	0.4757	0.4654
- Serum bicarbonate <28 mmol/l at follow-up	−3.6980	1.7085	0.0321
- Serum bicarbonate ≥28 mmol/l at follow-up	2.2055	1.0926	0.0454

Serum bicarbonate is used as a continuous variable and divided according to ≥ 28 mmol/l (knot). The HOMA-serum bicarbonate levels relationship changes for values of serum bicarbonate greater equal than 28 mmol/l

## Results

A total of 145 (57 % men) diabetic type 2, middle-age (65.5 ± 11.4 years) patients on oral antidiabetic medication were included in current analyses. At study inception, no significant differences in anthropometric, clinical and laboratory characteristics between subjects allocated to oral sodium bicarbonate or conventional therapy were observed (Table 1). In particular, treated subjects and controls exhibited similar renal function (mean creatinine clearance: 32 ± 14 ml/min and 35 ± 15 ml/min), serum bicarbonate levels (21.2 ± 1.9 mmol/l and 21.6 ± 2.0 mmol/l), fasting plasma glucose levels (149 ± 41 mg/dl and 151 ± 47 mg/dl), glycated hemoglobin (6.74 ± 1.0 % and 6.80 ± 1.4 %) as well as serum insulin levels (17.6 ± 6.1 mcIU and 19.0 ± 7.0 mcIU) (Table 1). Overall, basal HOMA-IR was 7.17 ± 2.4 and no difference between study groups was noted (median [Interquartile range (IQR)]: 6.4[5.5–7.9] and 6.4[5.5–8.2]; in the bicarbonate and control group, respectively). Of interest, only 4 (5,6 %) and 6 (8.1 %) subjects in the bicarbonate and control group had a HOMA-IR <5. Finally, at baseline HOMA-%B was also comparable between study groups (median [IQR]: 50.5 % [32.0–67.2 %] and 43 % [32.7–62.2 %]; in the bicarbonate and control group, respectively) (Table 1).

Table 1 shows patients’ antidiabetic and antihypertensive drugs. Moreover, Table 1 shows 25-OH vitamin D: the two groups did not show statistically significant differences of vitamin D blood levels (39 ± 10 versus 38 ± 10 ng/ml, in treated versus control, respectively)

Mean dose of oral bicarbonate administered was 0.7 ± 0.2 mmol/kg per each patient. At study inception there were no differences between the two groups in the use of oral antidiabetic drugs regarding number of pills, doses, and type of drugs (Table 1). No adverse affects were registered during oral bicarbonate administration.

At study completion, while no differences in renal function and blood pressure control were observed, a significant impact of oral sodium bicarbonate supplementation on serum bicarbonate levels (26.0 ± 2.0 vs 22.3 ± 1.9 mEq/l, in treated and control subjects, respectively) as well as diabetes control and management was apparent (Table 2). Specifically, HOMA-IR decreased in treated (p for within group comparison: 0.004) but not control subjects (p for within group comparison: 0.57) (median [IQR]: 5.9 [5.0–7.0] and 6.3 [5.3–8.2]; p for between groups comparison:0.01) (Fig. 1, Table 3). Similarly, HOMA-%B increased (p for within group comparison: 0.036) in the experimental group (p for within group comparison: 0.754) from a median [IQR] value of 50.5 % [32.0 – 67.2 %] to 60.5 % [43.5 – 70.2 %] while it was unchanged in the control group (median[IQR]: 43.0 [32.7 – 62.2] vs 45 [32.7 – 64.5] for baseline and follow-up, respectively; p value for between comparison at follow-up: 0.023) (Fig. 1, Table 3).

As documented in Fig. 2a and b, serum bicarbonate levels or changes were not linearly associated with insulin resistance. Improvement of serum levels of bicarbonate was associated with HOMA improvement only if metabolic acidosis over-correction (i.e., serum levels of bicarbonate greater than 28 mEq/l) did not occur. Indeed, a significant effect reduction (interaction test for treatement*serum levels of bicarbonate: *p* = 0.013) of oral bicarbonate supplementation on HOMA index occurred as serum bicarbonate rose (Table 4). To explore whether the effect on insulin resistance was due to the oral bicarbonate administration *per se* or metabolic acidosis amelioration, we performed splines regression analyses to account for the change in the relationship between serum bicarbonate levels and HOMA index according to metabolic acidosis correction (i.e., below or greater/equal than 28 mEq/l). As reported in Table 5, the benefit associated with metabolic acidosis correction disappeared when serum bicarbonate exceeded 28 mEq/l. Notably, when treatment allocation and serum levels of bicarbonate achieved were both forced into the spline regression model, treatment allocation lost statistical significance (*p* = 0.465) (Table 5), suggesting that metabolic acidosis correction rather than oral bicarbonate supplementation improves insulin resistance (Table 5).

## Discussion

Current findings suggest that metabolic acidosis is linked to insulin resistance in diabetic, Chronic Kidney Disease (CKD) patients and oral bicarbonate administration may correct metabolic acidosis that, in turn, improves insulin sensitivity in this population.

Insulin resistance (or reduced insulin sensitivity) is characterized by suboptimal biological responses of the liver, skeletal muscle and adipose tissue to normal amounts of insulin secreted [4, 5, 17–19]. Several biological processes such as glucose, lipid or protein metabolism as well as single hormonal effects such as glycogen synthesis or glucose oxidation may be affected in this condition [20, 21]. Several factors may contribute to insulin resistance in CKD. Visceral adipose tissue, diet, low physical activity, cigarette smoking, drugs (glucocorticosteroids, thiazide-like diuretics, beta-blockers) may all contribute to insulin resistance [22–24]. However, few lines of evidence also suggest that metabolic acidosis, that commonly complicates CKD, is implicated in suboptimal biological responses to insulin [6, 25].

Hence, metabolic acidosis represents a modifiable risk factor for insulin resistance and an attainable therapeutic target in CKD [4]. Indeed, metabolic acidosis may exert some detrimental effects at the cellular level inducing for example an intra-extracellular shift of cations and in different tissues such as bones and muscles as well as affect nutrition and metabolism [3, 6]. As part of CKD patients’ care, alkali such as sodium bicarbonate administration and/or low protein diet or diet rich in fruit and vegetables are commonly prescribed to avoid or correct metabolic acidosis. Preliminary evidence suggests that metabolic acidosis amelioration may attenuate CKD progression as well as hard outcome [17, 26–28].

Our results confirm and expand previous efforts [25, 29, 30] suggesting that metabolic acidosis correction by sodium bicarbonate administration improves insulin resistance without affecting the overall blood pressure control (Table 2). This is likely due to the better response to insulin of target organs (as suggested by the improvement of both HOMA-IR and HOMA-%B). In contrast with previous experiences [25, 29, 30], Ikizler and coworkers [31] recently failed to demonstrate an association between metabolic acidosis and insulin resistance in a cross-sectional, observational study of 42 patients with CKD stage 3–5. According to these findings, a reduced acid burden improved metabolic acidosis but not insulin sensitivity, measured via the hyperinsulinemic euglycemic clamp method [31]. Although we estimated rather than measured insulin resistance, our results suggest that, at least in diabetic CKD patients, over-correction of metabolic acidosis may also be detrimental since values of serum bicarbonate greater than 28 mEq/l are associated with decreased insulin sensitivity (Fig. 2). While Ikizler and coworkers [31] define metabolic acidosis as a dichotomous variable (i.e., serum bicarbonate level <22 mEq/l), we prospectively explored the association of serum bicarbonate as a continuous variable and insulin resistance over a broad range of values of serum bicarbonate (i.e., from 18 to 31 mEq/l). Current findings suggest that this association is non-linear (Fig. 2) and insulin sensitivity decreases for values of serum bicarbonate below 24 mEq/l and above 28 mEq/l. Of interest, accounting for the non-linear nature of the association also suggest that bicarbonate levels rather than sodium bicarbonate *per se,* is responsible for the effect on the HOMA index (Table 5).

In patients of treatment group assuming Biguanides (45 subjects), bicarbonate administration was higher (not significant) compared to other oral antidiabetic drugs (0.79 ± 0.4 mmol/kg).

Although further work is needed to validate these results in diabetic as well as non-diabetic CKD patients, the clinical relevance of these findings should be evaluated in light of the prevalence of insulin resistance and its associated complications such as hyperinsulinemia, hyperglycemia and hypertriglyceridemia [32]; the widespread use of sodium bicarbonate or alkali supplementation, low protein or vegetarian diet for CKD care [17, 33–40] as well as the safety and relative inexpensiveness of the treatment tested. Aside of confirming the link of bicarbonate and insulin resistance, current results also provide with some guidance for CKD patient care.

Our analyses suffer of a few limitations worth noting. We investigated the relationship of insulin sensitivity and metabolic acidosis in a subgroup of patients (diabetic patients on oral antidiabetic medications) randomized into the Use of Bicarbonate in Chronic Renal Insufficiency (UBI) study (NCT NCT01640119). This study aims at testing the impact of alkali administration and acidosis correction in diabetic and non-diabetic CKD patients on renal function decline. Although we analyzed a subgroup of patients, the analyses were carried out in the first 145 consecutive diabetic patients who completed at least 1 year of follow-up. This selection criterion as well as the random assignment to treatment at study inception are independent of the investigators’ beliefs and influences and we can argue that current findings are similar to a randomized clinical trial (RCT). The well balance of demographic, clinical and laboratory characteristics between groups, further corroborates this point. No power assumption or sample size calculation was performed in light of the exploratory nature of these analyses and the lack of similar data in this domain. Insulin resistance is calculated rather than measured. However, the HOMA test is widely accepted as a reliable and reproducible tool to assess insulin sensitivity in clinical and epidemiological studies [12–16, 41, 42].

## Conclusions

In conclusion, current results corroborate the notion that metabolic acidosis promotes insulin resistance and shed some light on the impact of sodium bicarbonate administration in CKD diabetic patients. Although further validation is mandatory, it seems that serum bicarbonate levels rather than the treatment used is relevant to restore insulin sensitivity. Finally, acidosis overcorrection (i.e., serum bicarbonate levels >28 mEq/l) should be avoided since, as metabolic acidosis, is associated with insulin resistance.

## Abbreviations

ASCVD: Atherosclerotic cardiovascular disease
CKD: Chronic kidney disease
DM2: Diabetic type 2 patients
ESRD: End stage renal disease
HOMA: Homeostatic model assessment
HOMA-%B: ß pancreatic cell function calculate by Homa test
HOMA-IR: Calculate insulin resistence by Homa test
IR: Insulin resistance
MA: Metabolic acidosis
NYHA: New York Heart Association
RAAS: Renin-Angiotensin-Aldosterone System
UBI: Use of bicarbonate in chronic renal insufficiency
